# Missed nursing care, non-nursing tasks, staffing adequacy, and job satisfaction among nurses in a teaching hospital in Egypt

**DOI:** 10.1186/s42506-021-00083-0

**Published:** 2021-07-20

**Authors:** Marwa Hammad, Wafaa Guirguis, Rasha Mosallam

**Affiliations:** 1 Ministry of Health, 97 El-Horeya Road, Qism Bab Sharqi, Wabour Al Meyah, Alexandria Governorate, Egypt; 2 High Institute of Public Health, Alexandria University, 165 El-Horeya Road, Al Ibrahimeyah Qebli WA Al Hadrah Bahri, Qism Bab Sharqi, Alexandria Governorate, Egypt

**Keywords:** Missed nursing care, Staffing adequacy, Non-nursing tasks, Egypt

## Abstract

**Background:**

Missed nursing care (MNC) has been linked to patient harm in a growing body of literature. However, this issue is still not adequately investigated in developing countries. The aim of the study is to measure the extent of missed nursing care, to identify its types, and to determine factors contributing to missed nursing care.

**Methods:**

A cross-sectional design was used. The study was conducted among 50 units at 1762-beds teaching Hospital in Alexandria that employs 1211 nurses in inpatient areas. A sample of 553 nurses were interviewed using the MISSCARE and the N4CAST survey. The MISSCARE survey measured the amount of missed nursing care (MNC) that was experienced on the last worked shift by each nurse. The N4CAST survey was used to collect data about level of non-nursing work carried out by nurses and the nurses’ job satisfaction.

**Results:**

The overall mean score for the missed nursing care was 2.26 ± 0.96 out of 5, with highest mean score attributed to “Planning” and lowest mean score attributed to “Assessment and Vital Signs” (2.64 and 1.96, respectively). Missed nursing care was significantly associated with number of patients admitted and cared for in the last shift and perceived staffing adequacy. Almost all non-nursing care tasks and most of satisfaction elements showed negative weak correlation with overall missed nursing care.

**Conclusion:**

Missed Nursing Care is common in study hospital which may endanger patient safety. MNC Missed Nursing Care is positively associated with nursing adequacy. There is no association between MNC and neither nurses’ job satisfaction nor non-nursing tasks. Nursing leaders should monitor missed nursing care and the environmental and staffing conditions associated with it in order to design strategies to reduce such phenomena.

## Introduction

The term “missed nursing care” (MNC), introduced in 2006 [1], has been defined as “any aspect of required patient care that is omitted (either in part or in whole) or delayed” [2]. In a systematic review of 42 studies, four studies concluded that MNC is a significant problem in hospitals; 55–98% of nurses reporting missing one or more items of required care during the time of assessment (frequently the last shift worked) [3]. The most frequently identified missed items were ambulating and turning patients, mouth care, feeding patients on time, comfort talk with patient and family, patient teaching, medication administration on time, and documentation [4–6].

Studies investigating reasons for MNC, as mentioned by nurses, concluded that inadequate labor resources was the most frequently cited reason, followed by material resources then communication. The most frequently reported items in labor resources were unexpected rise in patient volume and/or acuity and inadequate number of staff, those in material resources were unavailability of medications when needed, and unavailability of supplies and equipment, while the most common items reported in communication were unbalanced patient assignments, and breakdowns in communication with medical staff [4, 6–8].

Studies examining the relationship between nurse staffing and MNC, used different staffing measures, and reported inconsistent findings. Using the objective measures of hours per patient day (HPPD), registered nurse hours per patient day, and skill mix, one study conducted in 10 hospitals in the USA identified HPPD as the only significant predictor of MNC [9], while another study in 11 hospitals in the USA reported that none of the measures was a significant predictor [5], and a third study in two hospitals in the USA reported that these measures were not predictors of patient-reported MNC [10]. Another study comparing MNC in high- vs. low-staffing units in South Korea revealed that nurses working in high-staffing units had a significantly lower mean score of MNC than those in low-staffing units [11]. Nursing perceived level of adequate staffing was found to be a significant independent predictor in a study conducted in the USA, where those who perceived their staffing as adequate more often reported less MNC [4], while the same measure was not a significant predictor in another study conducted in eight hospitals in Iceland [12]. Four studies, using patient-to-nurse ratio as a measure of staffing, reported conflicting findings. Two studies, one conducted in 488 hospitals across 12 European countries and the other conducted in 10 acute care hospitals in the USA, concluded that higher patient-to-nurse ratio was a significant predictor of MNC [4, 13], while another study reported that a higher patient-to-nurse ratio was a significant predictor of less MNC in an Italian medical care setting [6], and a fourth study conducted in Lebanon revealed that the patient-to-nurse ratio was not an independent predictor of MNC [14].

Several studies identified nurses spending their time performing non-nursing tasks (NNT) as an important reason for MNC. NNT include all tasks that are not related to direct patient care or tasks not requiring professional nursing skills [15–17]. In a study conducted in 55 private hospitals and seven public hospitals in South Africa, nursing tasks left undone were related to three non-nursing tasks, namely, “delivering and retrieving food trays,” “routine phlebotomy/blood drawing for tests,” and “cleaning patients’ rooms and equipment” [17].

As regards the impact of MNC on nurses, a study conducted in 10 hospitals in the USA identified MNC as a significant predictor of intention to leave, but not of turnover [18]. Another study in the USA identified MNC as a significant predictor of job satisfaction; nurses who reported less MNC were more satisfied in their current position and occupation [19].

Literature search identified four Egyptian studies on MNC; all conducted in critical care units, and based on a small sample of nurses [20–23]. This research is a hospital-wide study to identify level and types of MNC and to determine factors contributing to missed nursing care, so that appropriate interventions can be developed to reduce care omissions and thus improve quality and safety of care.

## Methods

### Study design and setting

The study was conducted among 50 units at 1762 beds teaching hospital that employs 1211 nurses in inpatient areas. A cross-sectional design was used. The study population was nurses working in inpatient units who have been working in the hospital for a minimum of 3 months.

### Sample

The following assumptions were used to calculate the required sample size of nurses to estimate the mean of MNC score: 95% confidence interval for mean ± 0.1 and standard deviation of 1.2 [20]. The minimum required sample size is 553 nurses (1.96*1.2/0.1). Units were randomly selected from all hospital inpatient units, and all nurses who fulfilled the inclusion criteria in each selected unit were invited to participate using convenient sampling, until the required sample size was reached. Inclusion criteria were nurses working in inpatient units of the study hospital who have been working in the hospital for a minimum of 3 months.

### Data collection

A structured interview schedule was prepared to collect data from nurses about level of overall and specific elements of MNC, unit characteristics, nurse characteristics, nurse staffing measures, and nurse outcome measures.

The interview schedule consisted of two tools, the MISSCARE survey and the N4CAST survey. The MISSCARE survey [24] measures the amount of MNC that was experienced on the last worked shift by each respondent. It is composed of 24 items, belonging to four areas as follows: assessment and vital signs (8 items); interventions and individual needs (6 items); intervention/basic needs (7 items); and planning (3 items). For each item, respondents are asked to identify the frequency of care being missed using a 5-point Likert scale from never missed to always missed. The mean of all 24 items is used as a total score for the scale, and the potential range of scores is 1 to 5, with higher scores indicating more MNC. The questionnaire also collects data about work and demographic characteristics of nurses. The N4CAST survey [25] was used to collect data about level of NNT carried out by nurses. This section contains a list of nine tasks, and each nurse was required to indicate the frequency with which she/he performed each task during the most recent shift, on a 3-point scale of never, sometimes, and often (scored as 0, 1 and 2, respectively). The items for NNT were the following: delivering food trays, performing non-nursing care, arranging discharge referrals and transportation, phlebotomy, transporting patients within the hospital, cleaning patients’ rooms, filling-in for non-nursing services, obtaining supplies, and performing clerical duties. The N4CAST survey was also used to measure level of nurses’ job satisfaction. Each nurse was required to indicate the level of satisfaction with the current job on a 4-point scale of very dissatisfied, dissatisfied, satisfied, and very satisfied (scored as 1, 2, 3, and 4, respectively). The items for satisfaction were the following: work schedule flexibility, opportunities for advancement, independence at work, professional status, salary, educational opportunities, and annual sick and study leaves.

The study tools were translated to Arabic by the research team and then reviewed by a professional translator. The tool was assessed for face validity by a panel of experts. The questionnaire was piloted on 20 nurses to assure understandability and relevance of items. There were no changes in the tool and the 20 questionnaires were included in the sample.

### Statistical analysis

Data was entered and analyzed using SPSS (Statistical Package for Social Sciences) version 21. Descriptive statistics (frequencies, percentages, and means) were performed. T test was used for comparison of means between two groups and one-way ANOVA for comparison of means of more than two groups. The 5% level was used as a cut-off point value for statistical significance. Spearman rank-order correlation was used to assess the correlation between MNC and non-nursing tasks and nurses’ job satisfaction. Spearman rank-order correlation was selected as the data was not normally distributed.

### Ethical considerations

Approvals of Ethics Committee of High Institute of Public Health and the Director of Central Administration of Alexandria Hospitals were obtained. Verbal consent was obtained from participants in the study, who were assured of confidentiality and anonymity of information and that it will be used for research purposes only. The researchers declare no conflict of interest.

## Results

The majority of the study sample were females (94.4%), in the age category 30 < 50 years (60.6%), working in surgical units (60.6%), holding a nursing diploma degree (68.0%), and working as a “Practical Nurse” (66.5%).

As for workload of nurses at study hospital, the majority of nurses worked for more than 30 h per week (89.0%) and cared for more than 10 patients per shift (43.4%) with a mean of 11.94 patients. Highest percentage of the nurses had 0-5 patients admitted and discharged during the last shift (84.6% and 89.5%). As for perceived staff adequacy, around one-third of the sample perceived the staff to be adequate 50% of time and 16.1% perceived that the staff was never adequate.

The overall mean score for the MNC was 2.26 with highest mean score attributed to “Planning” and lowest mean score attributed to “Assessment and Vital Signs” (2.64 and 1.96, respectively). Item with the highest mean scores was attending “conferences” (2.84), whereas items with the least mean score was “IV site care,” “Vital signs assessed as ordered(1.74 ± 1.32),” and “Full documentation of all necessary data” ) (1.76±1.36) (Table 1).

**Table 1:** Mean scores of missed nursing care among inpatient nurses in a teaching hospital in Alexandria, Egypt

Elements of MISSCARE survey	Total (*n* = 553)
**Assessment and vital signs (overall)**	**1.96 ± 1.07**
Full documentation of all necessary data	1.76 ± 1.36
IV site care and assessment according to hospital policy	1.70 ± 1.31
Monitoring intake/output	1.90 ± 1.30
Vital signs assessed as ordered	1.74 ± 1.32
Focused reassessment according to patient condition	2.47 ± 1.54
Hand washing	1.99 ± 1.43
Bedside glucose monitoring as ordered	1.88 ± 1.38
Patient assessments performed each shift	2.26 ± 1.55
**Interventions and individual needs (overall)**	**2.19 ± 0.98**
Assessing effectiveness of medications	2.30 ± 1.44
PRN medication requests acted up on within 15 min	2.06 ± 1.41
Medications administered within 30 min before or after scheduled time	1.96 ± 1.28
Assist with toileting needs within 5 min of request	2.44 ± 1.40
Response to call is provided within 5 min	2.0 ± 1.34
Emotional support to patient and/or family	2.41 ± 1.47
**Intervention /basic needs (overall)**	**2.48 ± 1.17**
Ambulation three times per day or as ordered	2.72 ± 1.48
Turning patient every 2 h	2.44 ± 1.49
Mouth care	2.64 ± 1.56
Feeding patient when the food is still warm	2.58 ± 1.53
Patient bathing	2.65 ± 1.62
Setting up meals for patients who feed themselves	2.29 ± 1.58
Skin/wound care	2.01 ± 1.37
**Planning (overall)**	**2.64 ± 1.08**
Patient teaching	2.59 ± 1.36
Attending interdisciplinary care conferences whenever held	2.84 ± 1.42
Ensuring discharge planning	2.50 ± 1.52
**Overall missed care**	**2.26 ± 0.96**

Score (1–5) with higher score indicating higher MNC level

MNC was not significantly associated with any of the work and demographic characteristics except the unit where the ICU was the unit with the lowest mean score as compared to the medical and surgical units (1.91 versus 2.48 and 2.30, respectively) (Table 2).

**Table 2:** Association between overall missed nursing care and demographic and work characteristics

Characteristic	Overall MNC Mean ± SD	Test of Sig.^*^	p
**Sex**		1.15	0.24
Male	2.45 ± 0.84		
Female	2.24 ± 0.84		
**Age group (years)**		1.70	0.14
< 20	2.30 ± 0.88		
20 to < 30	2.25 ± 0.88		
30 to < 40	2.12 ± 0.86		
40 to < 50	2.27 ± 0.99		
≥ 50	2.43 ± 1.11		
**Unit**		14.31^*^	0.000
Medical unit	2.48 ± 0.77		
Surgical unit	2.30 ± 0.87		
ICU	1.91 ± 1.06		
**Professional qualification (highest degree)**		1.42	0.22
Nursing diploma (high school equivalent)	2.29 ± 0.94		
Nursing technician diploma	2.29 ± 1.19		
BSN (bachelor’s degree)	2.04 ± 0.75		
Postgraduate diploma	1.94 ± 0.78		
Master’s degree or more	2.70		
**Job title**		1.22	0.30
Head nurse (head of unit)	1.98 ± 0.90		
Nurse supervisor (BSN)	2.14 ± 0.78		
Nurse technician	2.29 ± 1.19		
Practical nurse (high school equivalent)	2.28 ±0.93		
**Shift worked**		1.80	0.14
Morning	2.32 ± 1.05		
Evenings	2.26 ± 0.92		
Nights	1.94 ± 0.65		
Rotates	2.20 ± 0.87		

^*^P value < 0.05

(T test was used for comparison of means between two groups and one-way ANOVA for comparison of means of more than two groups)

MNC was significantly associated with number of patients admitted and cared for in the last shift and perceived staffing adequacy. The higher the patients admitted and cared for the last shift, and the lower the perceived staff adequacy, the higher the overall MNC score (Table 3).

**Table 3:** Association between overall missed nursing care and staffing adequacy, workload, and intention to leave

Characteristic	Total (*n* = 553)		Overall MNC Mean ± SD	Test of Sig.^*^	p
	No.	%			
**No. of working hours/week**				0.69	0.48
≥ 30 h	492	89.0	2.17 ± 0.99		
< 30 h	61	11.0	2.26 ± 0.96		
**No. of patients cared for/last shift**				17.49^*^	0.00
0-5	213	38.5	1.97		
6-10	100	18.1	2.26		
> 10	240	43.4	2.49		
**Mean ± SD**	**11.94 ± 11.30**				
**No. of patient admissions/last shift**				4.32^*^	0.01
0-5	468	84.6	2.20		
6-10	78	14.1	2.54		
> 10	7	1.3	2.45		
**Mean ± SD**	**3.17 ± 2.74**				
**No. of patient discharges/last shift**				2.27	0.10
0-5	495	89.5	2.27		
6-10	49	8.9	2.23		
> 10	9	1.6	1.58		
**Mean ± SD**	**2.50 ± 2.81**				
**Perceived staffing adequacy**				7.88^*^	0.00
100% of the time	47	8.5	2.27 ± 1.00		
75% of the time	130	23.5	2.04 ± 0.81		
50% of the time	158	28.6	2.07 ± 0.80		
25% of the time	129	23.3	2.41 ± 1.10		
0% of the time	89	16.1	2.64 ± 1.03		
**Work retention plan**				1.65	0.09
Plan to leave work within 6 months	137	24.8	2.38 ± 1.15		
No plan to leave work within 6 months	416	75.2	2.21 ± 0.88		

^*^P value < 0.05

(T test was used for comparison of means between two groups and one-way ANOVA for comparison of means of more than two groups)

Unexpectedly, almost all non-nursing care tasks showed negative weak correlation with overall MNC. This means that the more the non-nursing tasks, the lower the overall MNC score (Table 4).

**Table 4:** Spearman rank-order correlations between overall missed nursing care and non-nursing tasks

Non-nursing task	Total (*n* = 553) Mean (SD)^a^	Overall MNC
**Delivering and retrieving food trays**	0.79 (0.81)	−0.133^*^
**Performing non-nursing care**	1.14 (0.70)	−0.017
**Arranging discharge referrals and transportation (including long-term care)**	1.43 (0.68)	−0.077
**Routine phlebotomy/blood draw for tests**	1.72 (0.53)	−0.041
**Transporting patients within hospital**	1.36 (0.65)	−0.085^*^
**Cleaning patient rooms and equipment**	1.45 (0.68)	−0.259^**^
**Filling in for non-nursing services and available on off hours**	0.96 (0.78)	0.027
**Obtaining supplies or equipment**	1.53 (0.63)	−0.085^*^
**Answering phones, clerical duties**	0.79 (0.81)	0.056

^*^Spearman correlation coefficient *P* value < 0.05

** Spearman Correlation Coefficient *P* value < 0.01

^a^Scores (0-2)

Most of satisfaction elements showed negative weak correlation with the overall MNC. This means that the higher the satisfaction, the lower the overall MNC score (Table 5).

**Table 5:** Spearman rank-order correlations between missed nursing care and satisfaction

Satisfaction dimension	Total (*n* = 553) Mean (SD)^a^	Overall MNC
**Work schedule flexibility**	2.49 ± 0.90	−0.136^**^
**Opportunity for advancement**	2.47 ± 0.92	−0.110^**^
**Professional status**	2.49 ± 0.96	−0.186^**^
**Salary**	1.82 ± 0.88	−0.103^*^
**Educational opportunities**	2.25 ± 0.91	0.015
**Annual leave**	2.26 ± 0.90	0.054
**Sick leave**	1.99 ± 0.92	−0.119^**^
**Study leave**	2.05 ± 0.91	−0.001
**Current job**	2.87 ± 0.85	−0.066
**Independence at work, be a nurse or technician**	2.83 ± 0.88	−0.095
**Team work and co-operation within department/unit**	2.89 ± 0.88	−0.079
**Overall**	2.40 ± 0.56	−0.170^**^

^*^Spearman correlation coefficient *P* value < 0.05

** Spearman Correlation Coefficient *P* value < 0.01

^a^Scores (1–4)

## Discussion

The overall MNC in the current study is much higher than a study comparing the MNC in the USA and Lebanon (2.26 for the current study versus 1.71 and 1.31 in the USA and Lebanon, respectively) [14]. Similarly, in another study conducted in a public hospital in South Korea, the overall MNC was 1.39 for high staffing units (7 patients per RN) versus 1.51 for low staffing units (17 patients per RN) [6]. However, the results were comparable to the overall mean scores in four public hospitals in Australia where the mean scores ranged from 2.02 in ICU units to 2.40 in the medical units [26].

The overall dimensions “assessment and vital signs” and “interventions and individual needs” had less missed opportunities when compared to the overall dimensions “basic needs” and “planning.” This result is consistent with the results of other studies [4, 7, 27]. Possible explanations for the MNC items is that those tasks that respondents perceived as important (e.g., vital sign and glucose monitoring) and which did not require teamwork were less frequently missed. On the other hand, care items, which may be perceived as less serious, although linked to patients’ outcomes, for example, mouth care and those that often require teamwork (such as ambulation) were more likely to be missed [7]. Another possible explanation is that least frequently reported elements of MNC as vital signs, monitoring intake/output, and glucose monitoring are routinely recorded in nursing documentation and hence recognized by others if the medical file was audited. Conversely, ambulation of patients, patients’ bathing, mouth care, and turning patients are nursing duties not routinely recorded in nursing documentation, and there is less opportunity for others to perceive this care as missed. Increased attention to these elements, including a refocus of existing documentation systems, is a necessity [27].

Hospital unit was significantly associated with the overall MNC score where the ICU had a lower overall MNC score when compared to the medical and surgical units (1.91 versus 2.48 and 2.30, respectively) (*P* < 0.000). This result is consistent with the results of a study conducted in eight hospitals in Iceland and another study conducted in four hospitals in Australia and among ICU units in two teaching hospitals in Egypt [7, 12, 20]. The better scores in the ICU can be explained by the fact that ICU has skilled nursing staff who hold at least bachelor degree in nursing. In addition, the nurse to patient ratio is usually 1:1 or 1:2 in ICU which reduce the workload on nurses and hence reduce the missed care.

Higher mean scores of MNC were significantly associated with increasing number of patients cared for and admitted in the last shift and nurses’ perceived inadequacy of staffing. Several studies conducted in the USA and South Korea, demonstrated the association between nurses’ workload and staffing adequacy and MNC [11, 12, 26, 28]. In other studies, staffing adequacy had a positive impact on reducing instances of failure to rescue, inpatient mortality, postoperative mortality, and length of hospital stay [29–31]. Thus, adequacy of staffing is of paramount importance in ensuring patient safety and reducing missed nursing care. Thus, it is recommended to use a staffing approach which accounts for individual patient variation in need as alternatives to, or in conjunction with, minimum staffing levels based entirely on patient volumes which is used in public hospitals in Egypt [32].

In the current study, only two non-nursing tasks correlated positively with missed nursing care: cleaning patients’ rooms and filling in for non-nursing services and available on off hours. Both correlations were weak and non-significant. This unexpected finding could be explained by the fact that nurses may consider all the other non-nursing tasks to be part of their workload. Studies pointed out that nurses accept performing non-nursing duties, because these roles are founded on organizational as well as individual patient relationships [33].

The current study showed transporting patients in hospital correlated negatively and significantly with certain missed nursing care elements. The rationale for this might be that when patients are transferred outside of the unit, it is crucial that all procedures are done before leaving the unit [17].

Overall satisfaction showed a negative weak significant correlation with overall MNC. This is consistent with the results of two studies conducted in the USA where nurses who perceived less MNC on the patient care unit where they work are more satisfied in their current position and occupation [19, 34]. This can have two possible explanations in the present study. First, is that when nurses see that the elements of nursing care are missed, they become more dissatisfied. This can be explained by the fact that people respond positively and with more motivation to do a good job when they witness their constituents benefiting from their efforts. In the case of nursing, unlike some other occupations, the providers have direct and many times immediate knowledge about the effect of the quality of their work on their patients. Thus, nurses are fully cognizant of the impact of missing care for their patients, and when the effect is negative [19, 35]. This was supported by a study conducted in the USA where each one unit increase in missed nursing care was associated with a 0.26 decrease in job enjoyment [36]. The second possible explanation is that the dissatisfied nurse is more prone to miss elements of nursing care. Further research should explore the causative relation between MNC and nurses’ satisfaction.

### Limitations

The current study addresses the concept of MNC in one of the largest teaching hospitals in Egypt. It sets a basis for further assessments and suggestions for improvement. Further research studying the link between MNC and adverse events is recommended.

The current study used subjective measures of missed care, with most relying on retrospective reports by nurses. Further studies using objective measures as direct observation of nursing care are recommended to evaluate the current study findings. The study was conducted in a teaching hospital which limits the generalization of the results to other settings as private, Ministry of Health and Insurance settings.

## Conclusions

The missed nursing care is relatively higher when compared to other settings. The dimensions which are frequently missed are “basic needs” and “planning.” The mean missed care was lower in the ICU as compared to the medical and surgical units. Nursing staffing adequacy and number of patients cared by nurse were factors associated significantly with higher score of missed nursing care. Non-nursing tasks and nurses’ job satisfaction showed negative weak correlation with the overall MNC.

## Data Availability

The data are available from the corresponding author on reasonable request.

## Abbreviation

MNC: Missed nursing care
